# Supplementary material for: Exploring near-optimal energy systems with stakeholders: A novel approach for participatory modeling
Source: iScience. 2026 Jul 9;29(8):116718. doi: 10.1016/j.isci.2026.116718 (PMC13380767; doi:10.1016/j.isci.2026.116718)
Supplement: Document S1. Figures S1–S7, Table S1, and Notes S1–S5 [file mmc1.pdf]

iScience, Volume *29*

Supplemental information

Exploring near-optimal energy systems with stakeholders: A novel approach for participatory modeling

Oskar Vågerö, Koen van Greevenbroek, Aleksander Grochowicz, and Maximilian Roithner

Supplementary Note 1 - Interactive interface

Below we provide figures of the different pages of the interactive interface, as they were shown to the participants in the study. An adaptable version of the interface is available on GitHub (`https://github.com/koen-vg/near-opt-interface`).

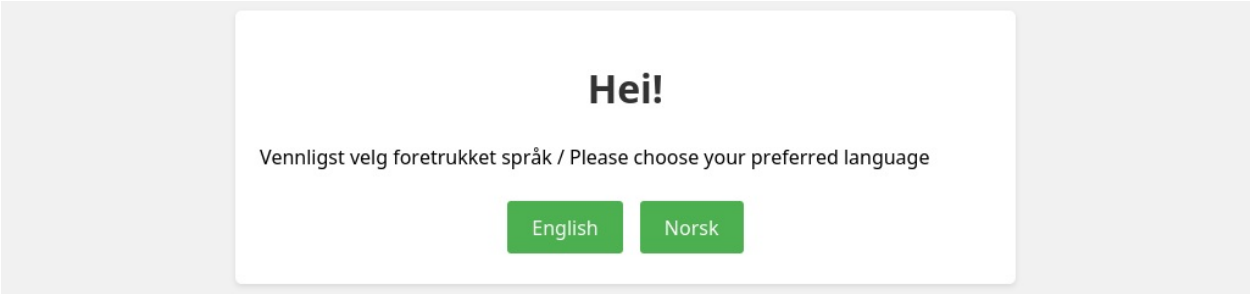

Figure S1: First page of interactive web interface. Participants are able to choose which language the rest of the interface is in. The following figures show the English options, but for every page there is also a Norwegian equivalent.

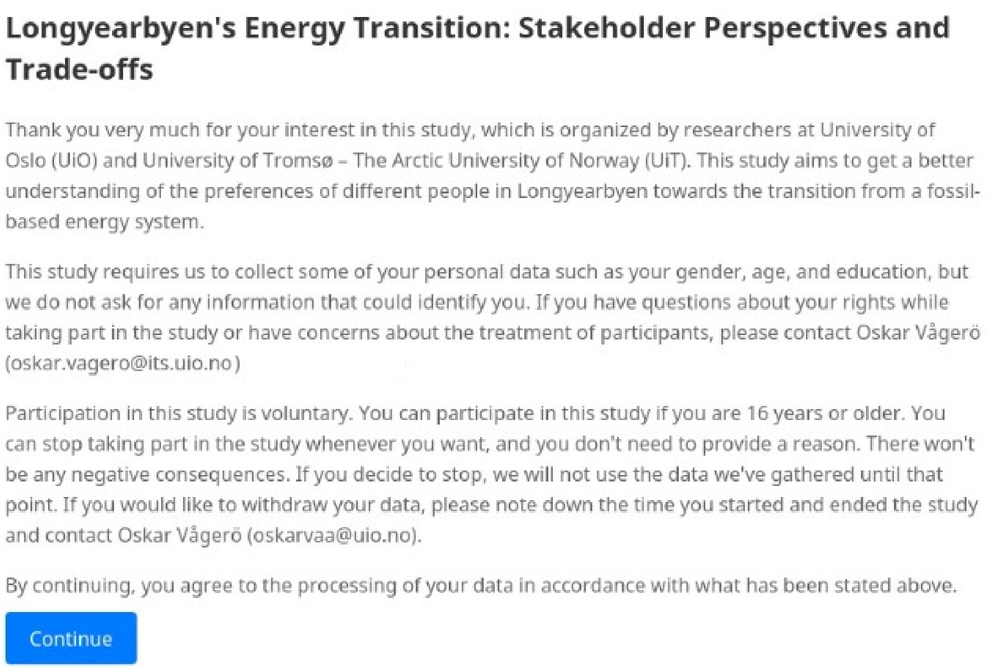

Figure S2: After selecting their language, participants are presented with some information on their participation in the project and data management of the project

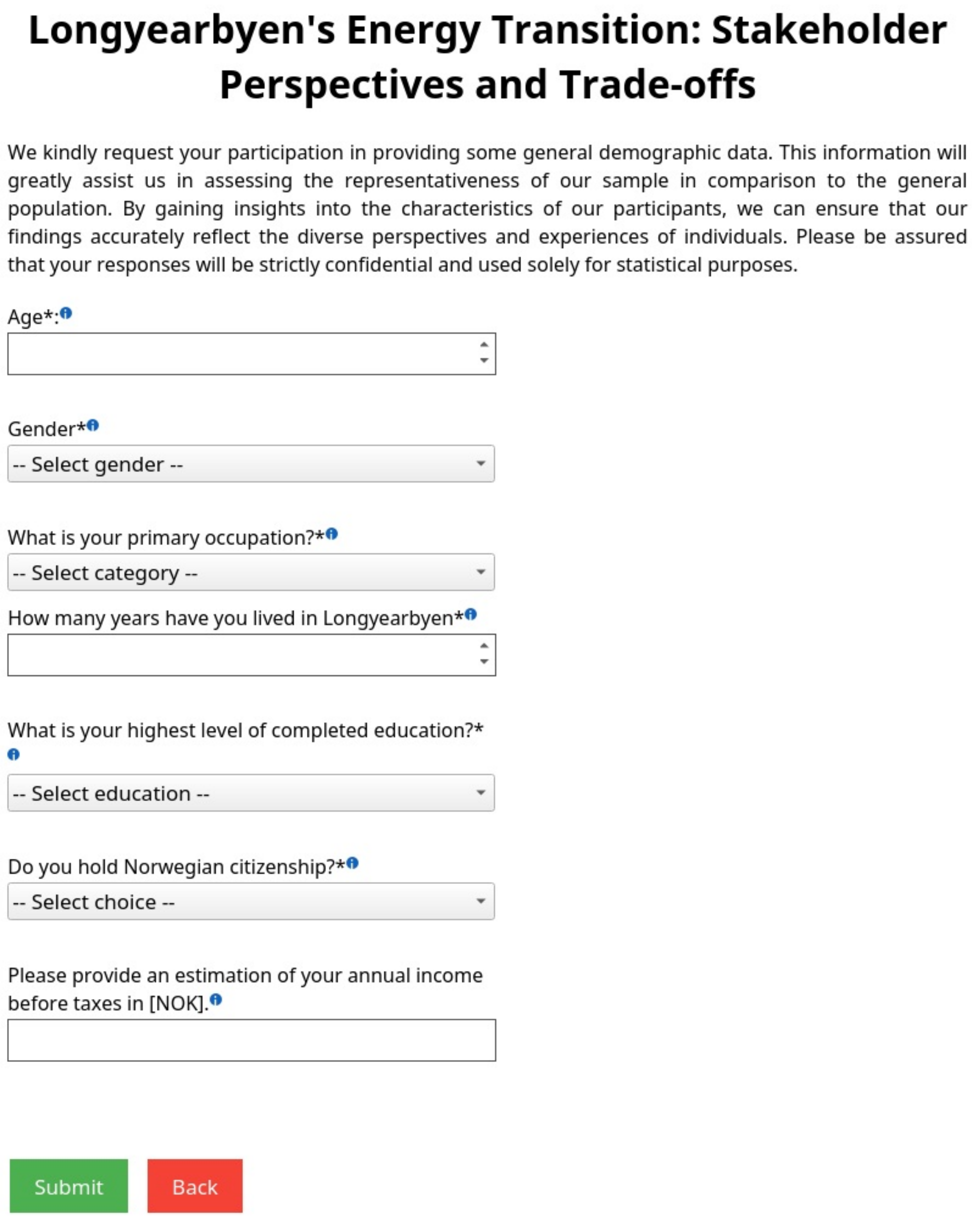

Longyearbyen's Energy Transition: Stakeholder Perspectives and Trade-offs

We kindly request your participation in providing some general demographic data. This information will greatly assist us in assessing the representativeness of our sample in comparison to the general population. By gaining insights into the characteristics of our participants, we can ensure that our findings accurately reflect the diverse perspectives and experiences of individuals. Please be assured that your responses will be strictly confidential and used solely for statistical purposes.

Age*:

Gender*

-- Select gender --

What is your primary occupation?*

-- Select category --

How many years have you lived in Longyearbyen*

What is your highest level of completed education?*

-- Select education --

Do you hold Norwegian citizenship?*

-- Select choice --

Please provide an estimation of your annual income before taxes in [NOK].

Submit Back

Figure S3: Participants are asked to include some information on themselves. Note that the question of income is optional.

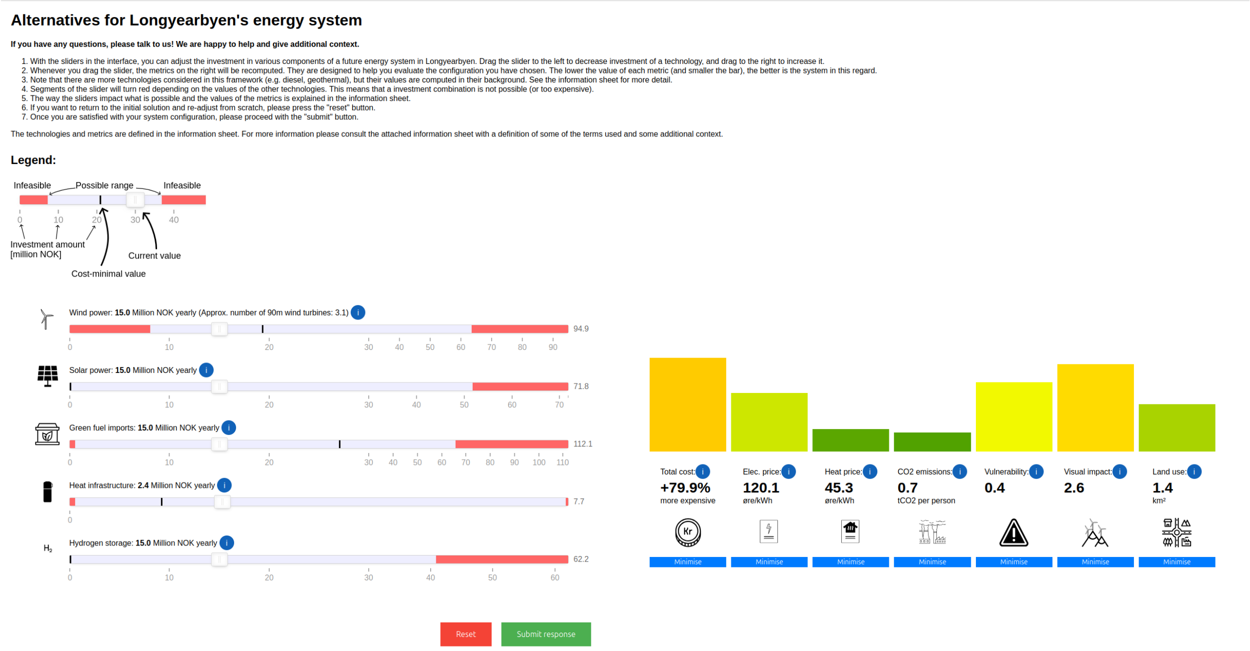

Figure S4: The main page of the interface. In the top, participants are given a brief introduction into how the interface works. Below and left is the sliders that the participants are able to move. The red zones that mark combinations that are infeasible is updated whenever a slider is moved. Below to the right are the metrics of the particular configuration. Participants are also able to minimise any of the metrics, from which they can further adjust their slider choices.

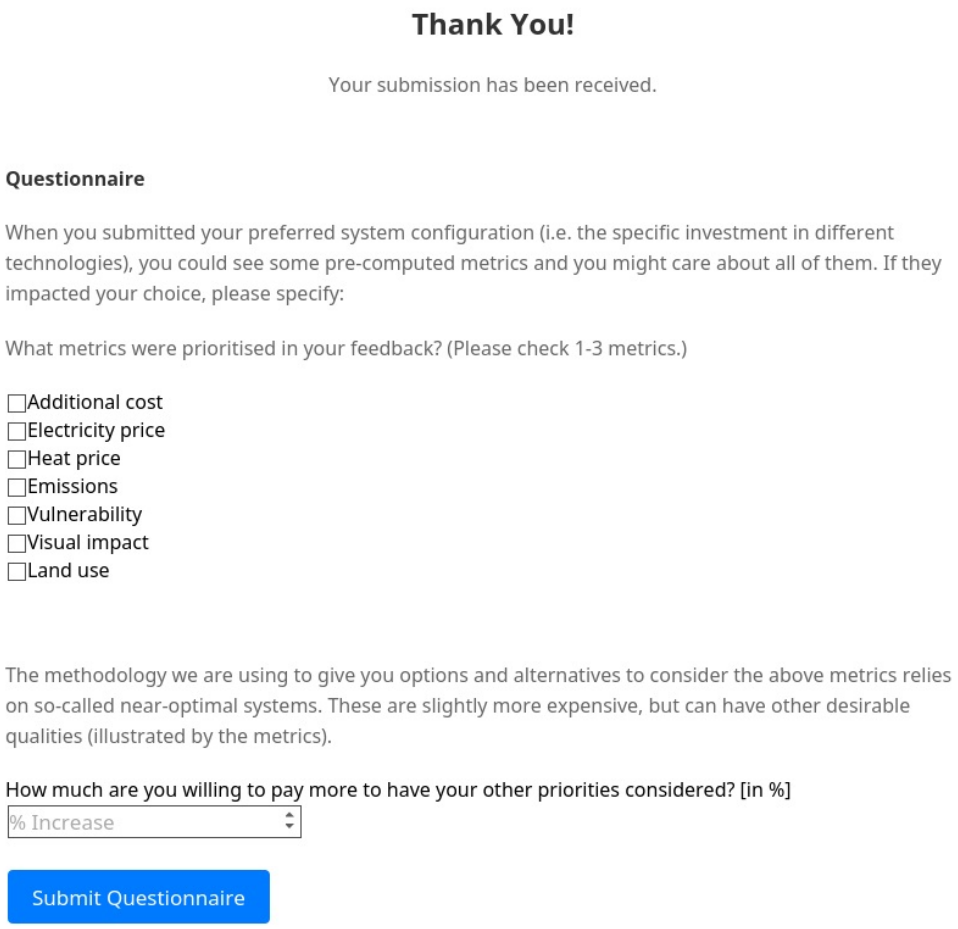

Figure S5: After submitting their combination of dimensions, participants are asked to state which of the metrics they prioritised and how much they consider themselves willing to pay to improve those (and other) considerations

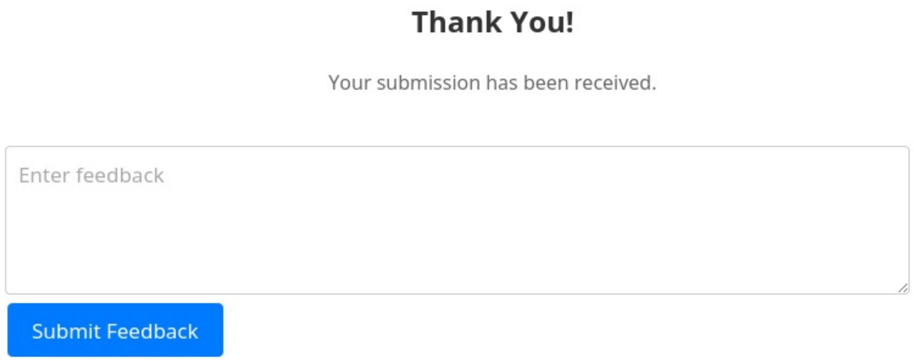

Figure S6: Lastly, participants are able to include written feedback in a free-text field.

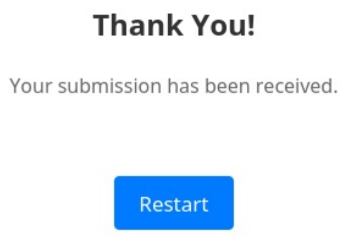

Figure S7: The last page of the interactive web interface

Supplementary Note 2 - Methodological differences from previous interactive interfaces

Previously participatory modelling exercises using some sort of interactive interface include the my2050 tool [1–3]), the Riskmeter [4–8]), COLLAGE [9] and the Portfolio-Building Computer Decision Tool [10].

The **my2050** tool was developed on the initiative of the UK Department of Energy and Climate Change to enable public engagement on energy futures [1]. There is an Excel-based version of the model that supports the online-version of the interactive interface. The model used for the interactive exercises has changed over the course of time and the current version may to some degree deviate from the published papers [1–3]. Users choose between four different levels of ambitions across 15 different categories, to reach net-zero energy systems. However, compared to the framework we propose and some of the other existing tool, the calculations and scenarios are based on a simulation model as opposed to an optimisation model. In the documentation of the Excel-version, it mentions that *"This is a simple model and cannot aim to fully capture the complexity of electricity capacity planning"*[11]. Supply and demand is balanced with a merit order of technologies' generation potential and with a simplified peak capacity test to ensure that demand can be met.

The **Riskmeter** is a tool developed by the Renewable Energy Systems group at the University of Geneva, Switzerland. The tool has been used in a number of publications and allows users to design an electricity portfolio for a selection of countries or the EU27. As stated in the description, the electricity portfolios are made on the basis of summing technology contributions, to achieve a set goal of meeting or even exceeding a projected future demand. The technology potentials which limit the amount of electricity a user can choose to generate is based on the energy systems optimisation model EXPANSE [12], which applies MGA. However, compared to the framework we present, the technology contributions are considered individually and are not accurate representations of the near-optimal feasible space, as each technology is considered individually. In contrast, the advantage of our approach is the integration of the full geometry of the near-optimal space with trade-offs being re-computed faithfully while the participants interact with the interface.

The **COLLAGE** tool [9] is an interactive planning tool used in stakeholder workshops in the Netherlands. It features a large-scale horizontal interactive display which participants can use to allocate wind power and ground-mounted/rooftop solar PV on a map. Users will see the resulting electricity generation while working towards a goal of achieving 12% of renewable electricity generation for a case study city. Similar to the Riskmeter, technologies are considered individually and complementarities between technologies as well as flexibility-needs for hourly balancing are not included.

Lastly, the **Portfolio-Building Computer Decision Tool** [10] is a computer-tool developed to facilitate informed decisions in energy planning. The tool features an Excel-based portfolio builder, which similarly to the other tools does not feature detailed temporal balancing of supply and demand. Instead, static capacity factors are applied for the different technologies, leading to generation potentials based on the utilisation rate set by users.

Overall, there are a few main methodological differences between previous exercises and our proposed framework. For one, while all participatory modelling exercises have an underlying model to some degree, the complexity and similarity to real-world processes and planning of previous tools are limited. In our proposed framework, building on the geometry of the near-optimal space, every system design selectable by the user is guaranteed to correspond to a feasible solution to the underlaying capacity expansion model with hourly temporal resolution. In contrast to the tools outlined above, the use of an energy system model (and MGA) allows us to take complex system interactions between renewables, storage and backup generation into account. Additionally, having the solutions tied to an energy system model means that the total system cost (investment and operational costs) can be accounted for accurately — a feature that is also only possible because the near-optimal solutions are computed using a model with high temporal resolution. This allows users of our proposed framework to effectively explore the large variety of solutions that are only marginally more expensive than the cost-optimal solution; a consequence of the near-optimal space being rather "flat" around the optimum [13]. Given the number of investment dimensions which the user can adjust independently, many different discrete scenarios cannot offer the same diversity of options and systematic overview for the participants.

Supplementary Note 3 - Information sheet for participants

Stakeholder preferences for energy under arctic conditions

Oskar Vågerö, Koen van Greevenbroek, Aleksander Grochowicz, Maximilian Roithner

March 8, 2024

Schedule

Table 1: Schedule

Day	Time	Location
Monday	09:00 – 13:00	UNIS
Monday	15:30 – 17:30	Svalbardbutikken
Tuesday	10:00 – 13:00	Fruene
Tuesday	15:00 – 18:00	Folkebiblioteket
Wednesday	11:00 – 14:00	Folkebiblioteket
Thursday	10:00 – 12:00	TBD
Thursday	15:00 – 17:00	Huskies

Introduction

The remote settlement of Longyearbyen is transitioning away from a fossil-based energy system, and while rough sketches of the desired end-state of the system exist, the suitability of different technologies are still under consideration. In this project, we wish to study the preferences of different local stakeholders and how they value different trade-offs with regard to an energy transition. To acquire this information, project participants will explore different energy system configurations in an interactive tool and submit their preferred energy system design. The interactive tool is based on energy system optimisation modelling (ESOM) with a version of the modelling framework *Python for Power System Analysis* (PyPSA).

Data collection

To study the preferences of stakeholders in Longyearbyen, we need to perform fieldwork and data acquisition.

Using the web-interface, we wish to study how different stakeholders value different trade-offs and how their preferred energy system.

Research

Deciding on a future energy system for Longyearbyen depends on utilising currently existing infrastructure as well as investing into new energy infrastructure and technologies. This has been studied in depth in the Norwegian government's "Energiplan Longyearbyen — Energiomstilling Longyearbyen 2023-2030" [Grøtte and Bøckman, 2023] where possible technologies and solutions are described. We are building on results and modelling conducted in this study to investigate the preferences of stakeholders and the public opinion on different aspects of the energy transition.

Often these problems can be described as a mathematical optimisation problem: essentially we find a solution which minimises system costs while ensuring that energy can be supplied at all times. The model

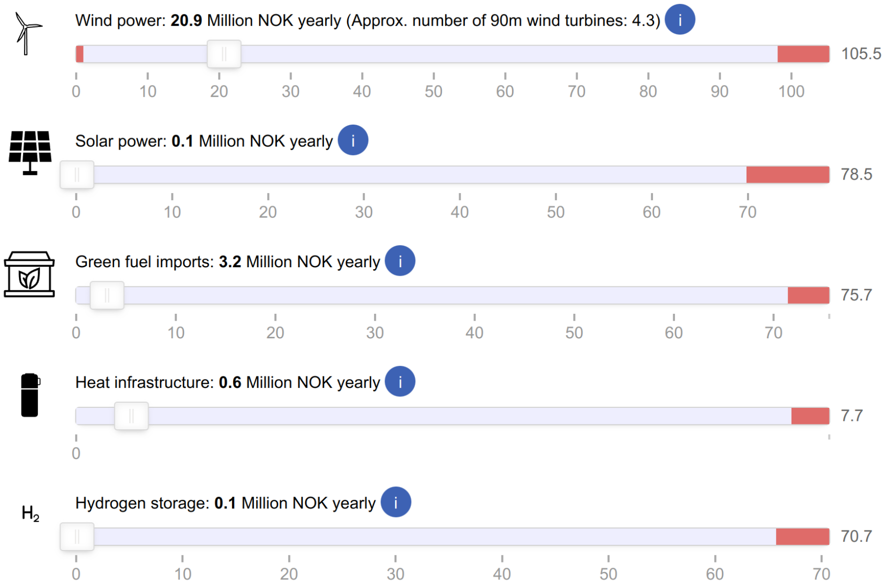

Figure 1: Screenshot of the sliders in the web interface

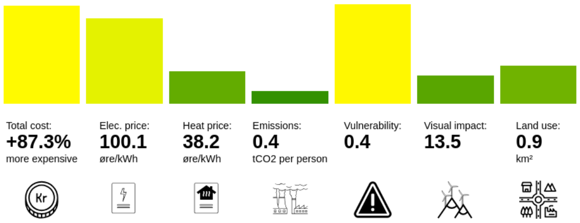

Figure 2: Screenshot of the computed metrics from the system selected in Figure 1

we use for this computes which technologies to invest in and how to operate the energy system in a (cost-)optimal fashion. Such "solutions" or results are usually presented by researchers to politicians and stakeholders as information and context to make efficient decisions. Policymakers in general want to keep system costs low, but might take other considerations into account. We are conducting this study in order to better understand what aspects are important to the local population and stakeholders in Longyearbyen.

For this, we have developed a framework which can describe a large set of alternatives and evaluate their quality under different aspects. The participants in this study can interact with the model to obtain energy systems which align with what they care about and can therefore deviate from the cost-optimal system. In the interface we have created for this study, the participants can freely adjust the levels of five system components (wind power, solar power, hydrogen, bioenergy, and heat storage) to obtain a possible future energy system that would reliably supply energy to Longyearbyen. These possible futures, that the participants can explore, are evaluated under different metrics (electricity costs, heating costs, visual impact, land use, emissions, supply security).

Research goals

Our study aims to find which decisions to achieve the energy transition in Longyearbyen are socially acceptable. We are interested in how different technologies are perceived by local stakeholders and what

metrics beyond cost-optimality are most relevant in the evaluation of possible energy systems. Lastly, we want to quantify if (and how much) stakeholders are willing to pay more if other objectives can be achieved.

Methodology

This study is based on a participatory modelling approach, which means that it is founded on a mathematical model which is combined with an interface through which participants can interact and express their preferences. The model we work with is an energy system optimisation model (based on the open-source modelling framework PyPSA), which can optimise investment and operational decisions for a future energy system in Longyearbyen. This model is owned by Longyearbyen Lokalstyret and Svalbard Energi AS and was developed by Multiconsult for the report "Energiplan Longyearbyen" [Grøtte and Bøckman, 2023] in 2023. By considering so-called near-optimal solutions (defined below) we can describe many more design alternatives than only one cost-minimal solution and therefore assess how different stakeholders prioritise various aspects of the energy transition and the impact on their community.

The participants of this study explore the large set of design alternatives in a web interface which differ in technology mix, costs, and are assessed by other metrics. Based on this large variety, of possible future systems, participants can decide which system best fits their preferences. We will analyse the results and preferences that the participants have anonymously submitted to our interface; we will inform Longyearbyen Lokalstyret about the results and insights. This research will be useful to other Arctic or remote settlements as well as to other communities which want to conduct their energy transition in a socially acceptable way.

Near-optimal modelling solutions

A near-optimal solution describes an alternative, feasible system design that is only slightly more expensive than the cost-optimal solution. Such near-optimal solutions can be desirable because they might outperform the cost-minimal solution with other qualities. Recent research on near-optimal solutions for energy systems has shown that these system configurations can vary widely without large cost differences [Grochowicz et al., 2023, van Greevenbroek et al., 2023]. Showcasing many near-optimal solutions can help stakeholders understand the flexibility in planning of systems; if we describe near-optimal spaces geometrically we can even systematically map out trade-offs. For a specified cost increase (also called slack level) of x%, we can say that all solutions cost roughly the same and we can focus on other qualities and metrics. As an example, we might be willing to have a slightly more expensive system to minimise a technology (e.g. solar power) or maximise other socially relevant metrics [Vågerö and Zeyringer, 2023].

Considered technologies

We have selected five "dimensions" that the participants can alter to reach their desired system configuration. All of those constitute the *investment* in this technology. These are

- total investment in **onshore wind power**,
- total investment in **solar power**,
- total investment in **hydrogen infrastructure**,
- total investment in **imported green fuels** and
- total investment in **heat storage**.

By dragging the sliders, the participants can change the investment value of one dimension at a time and this determines the admissible values of all other dimensions (impossible values are marked in red). These values come from tens of thousands of pre-computed near-optimal solutions to the model representation of Longyearbyen's energy system. The representation contains more technologies and system components than those with a slider such as diesel, ammonia, battery storage and geothermal energy.

For a more detailed system description see Figure 3.

Metrics

To evaluate the system configurations among which the participants can state their preferences, we have selected seven metrics:

- cost increase compared to the optimum
- electricity price,
- heat price,
- emissions,
- visual impact,
- land use and
- vulnerability.

We have chosen these metrics according to considerations that policymakers have to take into account: cost efficiency of the system, affordability (of electricity and heat), compatibility with climate targets (via emissions, but also to showcase whether Longyearbyen can be a role model for other Arctic settlements), social acceptance (visual impact, land use [Chen et al., 2022, Sasse and Trutnevyte, 2020]) and the vulnerability of the energy system. Since Longyearbyen is an isolated, off-grid community, it is vulnerable to weather variability and could become dependent on imports (in contrast to its past reliance on coal). Moreover, as the system needs to be self-reliant, an inclusion of too many technologies could be another risk factor. Lastly, we consider some heat storage (to avert infrastructure damage if heat supply were severed) to be essential to increasing the resilience of the community to unforeseen circumstances.

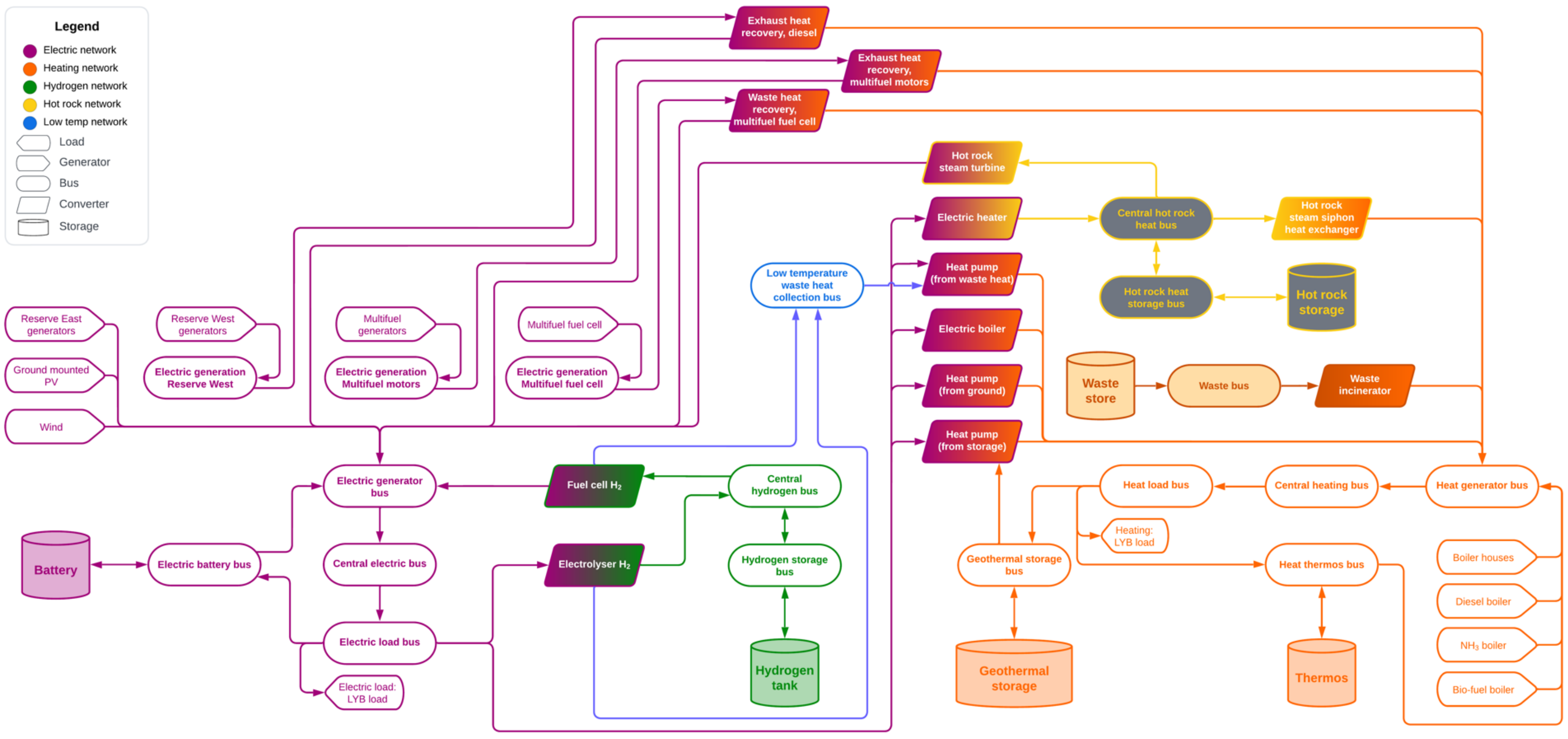

Figure 3: Illustration of how the energy system in Longyearbyen is modelled with PyPSA. Taken from Energiplan Longyearbyen [Grøtte and Bøckman, 2023].

Technologies (with specified slider)	Costs per energy produced (MWh)	Electricity price	Heat price	Emissions	Visual impact	Land use
Wind power	+	M	*	+	-	-
Solar power	+	M	*	+	+	-
Hydrogen infrastructure	M	M	*	+	+	*
Imported green fuels						
- Bioenergy (Pellets + Biogas)	o	M	*	+	+	-
- Ammonia	-	M	M	+	+	-
Heat storage	M	*	M	+	+	o
**Other technologies (without a slider)**						
Diesel	-	M	M	-	+	o
Battery storage	M	M	*	+	+	o
Geothermal energy	M	M	M	+	+	o

Technologies (with specified slider)	Vulnerability: Dependency on weather 20%	Vulnerability: Dependency on imports 50%	Vulnerability: Complexity of system 10%	Vulnerability: Back-up heat storage 20%
Wind power	-	+	o	*
Solar power	-	+	+	*
Hydrogen infrastructure	+	+	o	*
Imported green fuels				
- Bioenergy (Pellets + Biogas)	+	-	o	*
- Ammonia	+	-	o	*
Heat storage	+	*	o	+
**Other technologies (without a slider)**				
Diesel	+	-	o	*
Battery storage	+	+	o	*
Geothermal energy	+	+	o	*

Symbol	Meaning
*	Not applicable or missing data
M	Model output (system-dependent)
-	Strong negative impact
-	Negative impact
o	Weak negative impact
o	Very weak negative impact
+	Weak positive impact
+	Positive impact (or no impact at all)

Figure 4: Impact of different technologies on the metrics.

Unit		NOK/MWh	NOK/MWh	tCO_2	MW_wind	m^2
**Technologies (with specified slider)**	**Costs per electricity produced (kWh)**	**Electricity price**	**Heat price**	**Emissions**	**Visual impact**	**Land use**
Wind power	0.24 NOK	M	*	0	1	18000 (a1)
Solar power	1,06 NOK	M	*	0	0	50505 (a1)
Hydrogen infrastructure	M	M	*	0	0	*
Imported green fuels						
- Bioenergy (Pellets + Biogas)	2,37 NOK (heat: 0.47 - 1.19 NOK)	M	*	0	0	12,65 (indirect; b)
- Ammonia	3,03 NOK (heat: 1,51 NOK)	M	M	0	0	2,28 (indirect; b)
Heat storage	M	*	M	0	0	1,556 (a2)
**Other technologies (without a slider)**						
Diesel	3,35 NOK - 4,30 NOK (heat: 1,59 NOK)	M	M	0.254	0	25 (a1)
Battery storage	M	M	*	0	0	6.25 (a2)
Geothermal energy	M	M	M	0	0	1,556 (a2)

Unit	% Share of VRE	in [0,1] **		
		Vulnerability		
**Technologies (with specified slider)**	**Dependency on weather**	**Dependency on imports**	**Complexity of system**	**Back-up heat storage**
	20%	50%	10%	20%
Wind power	c	d	e	*
Solar power	c	d	e	*
Hydrogen infrastructure	c	d	e	*
Imported green fuels				
- Bioenergy (Pellets + Biogas)	c	d	e	*
- Ammonia	c	d	e	*
Heat storage	c	*	e	f
**Other technologies (without a slider)**				
Diesel	c	d	e	*
Battery storage	c	d	e	*
Geothermal energy	c	d	e	*

*	Not applicable or missing data
M	Model output (system-dependent)
**	
a1	per MW, Chen et al., 2022
a2	per MWh, Chen et al., 2022
b	per MWh, indirect land use from where it is imported, Sasse et al., 2020

Value for vulnerability is computed as 1 - (0.2 * c + 0.5 * d + 0.1 * e + 0.2 * f)

c	1 - share_vre
d	1 - share_imported_energy
e	1 - (1/6) * #{tech > 1 MW}
f	Cap in GWh (normalised to 1 corresp. to 4 GWh, which is enough to cover heat demand for two weeks in winter, and a month in summer)

Figure 5: Details on how the metrics are computed.

Limitations

Although we are interested in understanding choices and priorities of stakeholders as well as possible, we are forced to make some assumptions and simplifications in the model and study set-up.

The number of sliders (to control the investment in the different technologies) is limited in order to enhance the user experience, but also by computational constraints in the methodology. We have chosen five (groups of) technologies that are considered relevant in the context of Longyearbyen's energy system.

Similarly, we focus on a limited number of metrics to evaluate the different system configurations that participants can interact with. The selection is based on the explicit goals defined in 2022's Svalbardbudget as well as the development plan of Longyearbyen Lokalstyret. Some metrics that we consider relevant are inconclusive in the literature or difficult to quantify.

The energy transition in Longyearbyen is considered important, since it can serve as a blueprint for other Arctic or remote communities, which want to reduce emissions of their off-grid energy systems. Therefore, a solution relying heavily on local generation of renewable power (and possibly different storage technologies) can be viewed as being more valuable than importing green fuels with energy generated elsewhere — thus exporting the possible issues and conflicts elsewhere. Such considerations are beyond the scope of this study, as it is difficult to assess in advance which solutions might be applicable for other communities.

The energy system optimisation model we apply investigates single components (as illustrated in Figure 3) but for computational reasons, does not map out investments in solar and wind power spatially explicitly. This makes it difficult to assess the impact on nature, biodiversity, and visuals precisely and is beyond the scope of this study. Neither of the relevant locations for wind power fall within any of the nature reserves for birds[1]. Thus we decide to represent the impact on nature by land use (also indirect if green fuels are imported, e.g. from Northern Norway or elsewhere) and the visual impact by the installed capacities of wind power, which is a contentious issue in mainland Norway. Furthermore we can only take certain effects indirectly into account, such as the costs and vulnerability caused by a system consisting of many different components.

All the system configurations that the participants can interact with are based on a consistent set of assumptions ("scenario"). For more details we refer to the Energiplan Longyearbyen [Grøtte and Bøckman, 2023] which includes modelling results prepared by Multiconsult. We assume the current system now except that the coal mine (Gruve 7) is shut down — population and consumption patterns are assumed to be constant. This means that we do not include energy efficiency improvements (which are set out to lead to reductions of 30%) or electrified transport. The existing components are diesel boilers, batteries, the district heating system and the electricity grid as is. We assume that there is a CO_2 price of 2000 NOK/tCO_2, which affects the fuel costs of diesel.

Further questions

Who is responsible for the research project? The University of Oslo is responsible for the project. University of Tromsø – The Arctic University of Norway is a project partner.

Why have you been included in the study? For this project, participants are self-recruited in public spaces in Longyearbyen. We are interested in your opinion in this matter as a citizen of Longyearbyen. Ideally, the participants will be representative of the population.

What does the project mean for you? With the interactive tool you have on the computer in front of you, we want to learn how you value different factors in a future energy system in Longyearbyen. You will be able to change different dimensions of the energy system, such as from which energy sources electricity should be generated and see how your choices influence a few impact categories, such as visual impact, biodiversity impact and electricity prices. There are no right or wrong answers, and we are interested in your opinion. With the results, we want to better understand how to balance such impacts.

Can I object to my participation? You can object to being included in this research project at any time, and you do not have to give a reason. All your personal data will then be deleted. There will be no negative consequences for you if you choose to object.

[1] https://www.sysselmesteren.no/kart-og-gps/temakart/naturvernomrader/fuglereservat/

What happens to my data? We will only use the information about you for the purposes we have described in this letter. We treat the information confidentially and in accordance with the privacy regulations. Only project members at University of Oslo and University of Tromsø will have access to the data. Data collected is anonymous and will not include your name or other directly identifiable information. To minimise the risk that data collected in this study could be used to identify you, due to the small population of Longyearbyen, results will only be presented at an aggregate level.

What happens to your personal data at the end of the research project? The information will be anonymised when the project ends, which according to the schedule is 31.12.2025. At the end of the project, all individual data input will be deleted and only the aggregated results will be kept.

What gives us the right to process personal data about you? We are processing information about you because the research project is considered to be in the public interest, but you have the right to object if you do not wish to be included in the project. On behalf of the University of Oslo, Sikt – Norwegian Agency for Shared Services in Education and Research, has assessed that the processing of personal data in this project is in accordance with the privacy regulations.

What are my rights? As long as you can be identified in the collected data, you have the right to:

- object,
- access the personal data registered about you,
- have personal data about you corrected/rectified,
- have personal data about you deleted, and
- file a complaint with the Norwegian Data Protection Authority regarding the processing of your personal data.

For questions regarding this study or if you would like to know more or exercise your rights, please contact:

- Project leader **Oskar Vågerö** - oskarvaa@uio.no
- Our data protection officer **Roger Markgraf-By** - personvernombud@uio.no

If you have questions related to the assessment of this project by Sikt's data protection services, contact Data protection services via e-mail (personverntjenester@sikt.no) or by phone at: +47 73 98 40 40.

Glossary and facts about the energy system in Longyearbyen

- **Current situation:** Until 2023, Longyearbyen was powered by a coal power plant which lead to annual emissions of 70 000 tCO_2 (ca. 27 tCO_2 per capita only for electricity and heat supply, compared to total per capita emissions of 7.5 tCO_2 in Norway or 3.7 tCO_2 in Sweden). The coal power plant was replaced by a diesel power plant last year. Annual demand for electricity is approx. 32 GWh (disregarding Gruve 7) and heating demand is approx. 74 GWh.
- **Emissions and climate targets:** The current Norwegian climate target for 2030 is a reduction of greenhouse gas emissions by 55% (compared to 1990) and carbon net-neutrality by 2050 (in line with the Paris agreement). This means that we must not emit more greenhouse gases that our planet can remove from the atmosphere.
- **Energiplan Longyearbyen:** The Norwegian government (in the Svalbard budget 2021-2022) commissioned an energy plan that Longyearbyen Lokalstyret presented in early 2023. It describes the current system and possible futures that are in line with the Norwegian national climate targets for 2030 (reducing greenhouse gas emissions by 55%) and 2050 (carbon net-neutrality). For more details we refer to the Energiplan [Grøtte and Bøckman, 2023].
- **Energy and power:** *Energy* and electricity are usually denoted in kWh (kilowatt-hours) or MWh (megawatt-hours). 1 kWh is the amount of electricity that a typical light bulb with 10 Watt consumes during 100 hours. 1 kWh is also the unit for which the electricity price to consumers are defined. An average Norwegian citizen (on the mainland) consumes 7 000 kWh electricity per year[2]. *Power* is

[2] https://www.ssb.no/energi-og-industri/energi/artikler/hva-er-gjennomsnittlig-stromforbruk-i-husholdningene

usually denoted in kW (kilowatt) which also denotes the unit of peak (or maximum) capacity that a power plant can operate at. If the peak capacity of a plant is 1,000 kW, then in its peak hour it could produce 1,000 kWh (1000 kW times 1 hour).

- **Energy system:** An energy system combines various components which supply energy to cover energy service demand (such as heating, light). In this context, we look at electricity and heat as different energy carriers. By considering technologies that can generate or store energy, it can be ensured that all energy demand (for heating, light, cooking, industry, transportation etc.) in Longyearbyen can be met at all times.
- **Energy system optimisation model:** An energy system optimisation model describes the physical components of an energy system in a mathematical model. This makes it possible to explore different system configurations before building energy infrastructure in reality. Usually, an energy system optimisation model is formulated as an optimisation problem in which total system costs — consisting of infrastructure expansion decisions and operation decisions — are minimised. Subject to some simplifications, models provide valuable information to policymakers and planners on how an efficient system can be implemented. In this study, we present many alternatives to cost-minimal solutions which can be considered under different priorities and objectives.
- **Heat storage:** Heat can be stored over longer time periods in pits or tanks; excess heat produced can this way be utilised at later stages when it is needed. It provides additional security to the vulnerable infrastructure in the severe Arctic climate.
- **Hydrogen storage/infrastructure:** Hydrogen can be used to store excess electricity over longer time periods than batteries. Electricity is converted to hydrogen with an electrolyser (which splits water into hydrogen and oxygen) and if needed, fuel cells concert hydrogen (and oxygen) back into electricity (and water vapour). The round-trip efficiency is much lower than for batteries.
- **Imported green fuels:** In this model, it is possible to import *green ammonia* (in the future produced e.g. with excess electricity in Northern Norway) or *bioenergy* (either as biogas or pellets) and use it to provide energy to the system.
- **Interface:** The given interface combines the preferences of the study participant with the pre-computed alternatives generated by the energy system optimisation model. It allows the users to interact with realistic values and metrics that their chosen energy system design revealing the interactions of different decisions and technologies.
- **Land use:** Building energy infrastructure naturally occupies areas that could otherwise be used for various purposes. Different technologies require different amounts of infrastructure. For example, onshore wind occupies a rather small area for the construction itself, but requires access roads, especially during construction, to transport components to the location.
- **Near-optimal solutions and slack:** A near-optimal solution is a system configuration that is more expensive (by the cost slack, e.g. 5%) than the cost-minimal solution to the model. Near-optimal solutions can depict alternative system designs or have other, more desirable qualities, and are a useful basis for stakeholder involvement.
- **Participatory modelling:** Participatory modelling describes the involvement of stakeholders to interact with models which are used in decision processes. In this context, the participants can state their preferences in the interface and see the impacts that the model computes for the chosen system configuration. Similar interfaces have been developed in other contexts: for instance Riskmeter[3] [Xexakis and Trutnevyte, 2022], model.energy[4] and calliope explore[5] [Pickering et al., 2022].
- **PyPSA:** PyPSA[6] is an open source toolbox for simulating and optimising modern power and energy systems. In this study, we use PyPSA to explore different futures for Longyearbyen's energy system.
- **Technologies:** The participants can interact (through sliders in the interface) with five technologies to configure their preferred system design by choosing the level of investment of each technology.

[3] https://riskmeter.ch
[4] model.energy
[5] https://explore.callio.pe
[6] https://pypsa.org/

- **Visual impact:** For the estimation of visual impact, we consider mainly onshore wind to have an impact.
- **Vulnerability:** Off-grid communities, especially in the Arctic, are particularly vulnerable to severed supply of energy, which calls for different measures to enhance resilience. In this context, we separate the vulnerability in four categories: dependency on weather variability, dependency on imports, complexity of system and the scaling of back-up heat storage (whose existence and size boosts resilience).

References

[Chen et al., 2022] Chen, Y.-k., Kirkerud, J. G., and Bolkesjø, T. F. (2022). Balancing GHG mitigation and land-use conflicts: Alternative Northern European energy system scenarios. *Applied Energy*, 310:118557.

[Grochowicz et al., 2023] Grochowicz, A., van Greevenbroek, K., Benth, F. E., and Zeyringer, M. (2023). Intersecting near-optimal spaces: European power systems with more resilience to weather variability. *Energy Economics*, 118:106496.

[Grøtte and Bøckman, 2023] Grøtte, T. and Bøckman, R. (2023). Energiplan Longyearbyen - Energiomstilling Longyearbyen 2023-2030. Technical report.

[Pickering et al., 2022] Pickering, B., Lombardi, F., and Pfenninger, S. (2022). Diversity of options to eliminate fossil fuels and reach carbon neutrality across the entire European energy system. *Joule*, 6(6):1253–1276.

[Sasse and Trutnevyte, 2020] Sasse, J.-P. and Trutnevyte, E. (2020). Regional impacts of electricity system transition in Central Europe until 2035. *Nature Communications*, 11(1):4972.

[Vågerö and Zeyringer, 2023] Vågerö, O. and Zeyringer, M. (2023). Can we optimise for justice? Reviewing the inclusion of energy justice in energy system optimisation models. *Energy Research & Social Science*, 95:102913.

[van Greevenbroek et al., 2023] van Greevenbroek, K., Grochowicz, A., Zeyringer, M., and Benth, F. E. (2023). Enabling agency: Trade-offs between regional and integrated energy systems design flexibility.

[Xexakis and Trutnevyte, 2022] Xexakis, G. and Trutnevyte, E. (2022). Model-based scenarios of EU27 electricity supply are not aligned with the perspectives of French, German, and Polish citizens. *Renewable and Sustainable Energy Transition*, 2:100031.

Supplementary Note 4 - Supporting statistics in figures

Table S1: Statistical dispersion of data presented in Fig 5.

Category	Priority	Q1	Median	Q3
Additional costs	Yes	0.287	0.633	0.885
	No	0.762	0.994	1.167
Electricity price	Yes	981	1193	1421
	No	1036	1222	1454
Heat price	Yes	296	462	564
	No	377	458	578
Emissions	Yes	106	624	1744
	No	72	1416	2803
Vulnerability	Yes	0.319	0.360	0.457
	No	0.324	0.374	0.458
Visual impact	Yes	2.200	2.721	2.978
	No	2.637	2.881	3.175
Land-use	Yes	1.146	1.439	1.751
	No	1.351	1.620	1.922

Supplementary Note 5 - Example participant

To exemplify how the interface can be used and explored from a user-perspective, we can imaging an example participant who is a researcher who cares about low carbon emissions and is worried about the energy system being vulnerable.

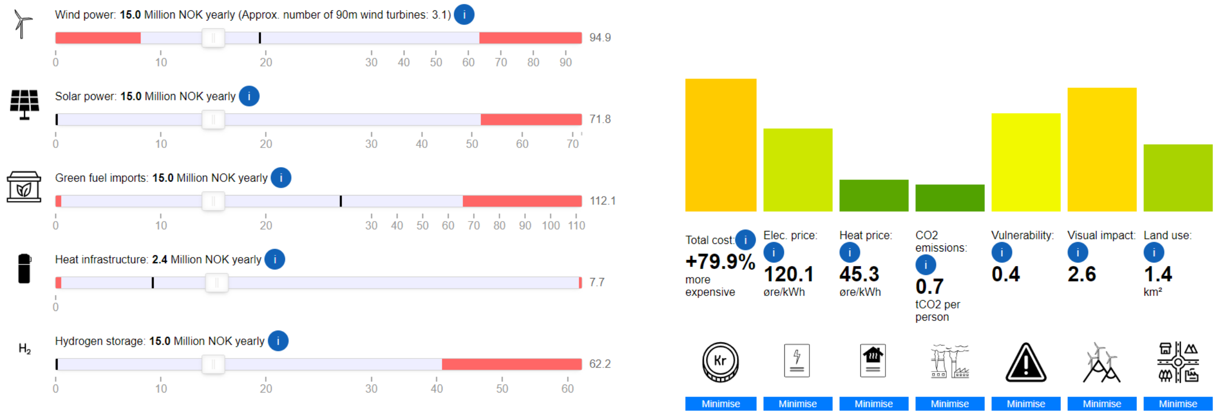

Exploring the interface, the participant starts by identifying the least-cost solution, which can be found by pressing the minimise button for total system cost.

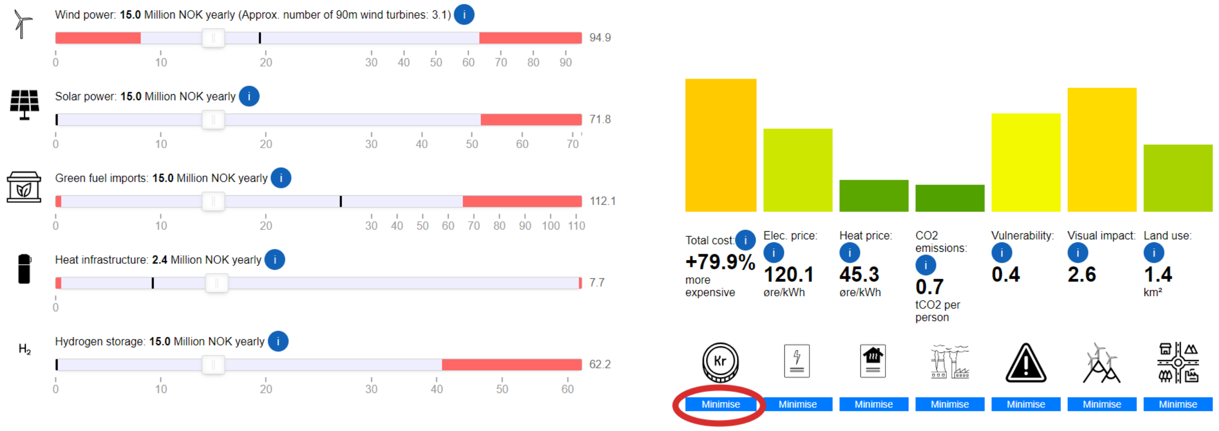

This results in a system with a fair amount of wind power and import of green fuels as well as some heat infrastructure.

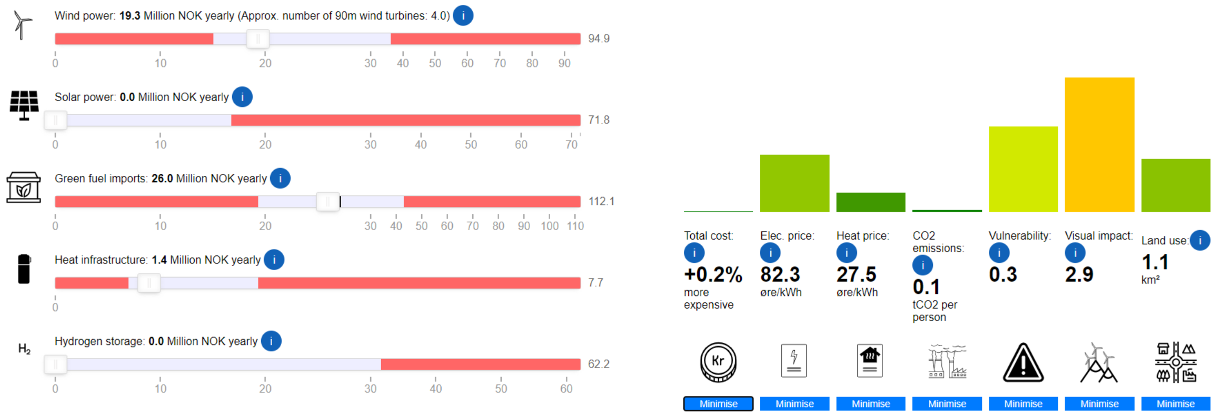

However, as mentioned in the beginning, our participant is very concerned about both emissions and vulnerability. As such, they choose to explore alternatives to the least-cost solution by minimising the CO_2 emissions.

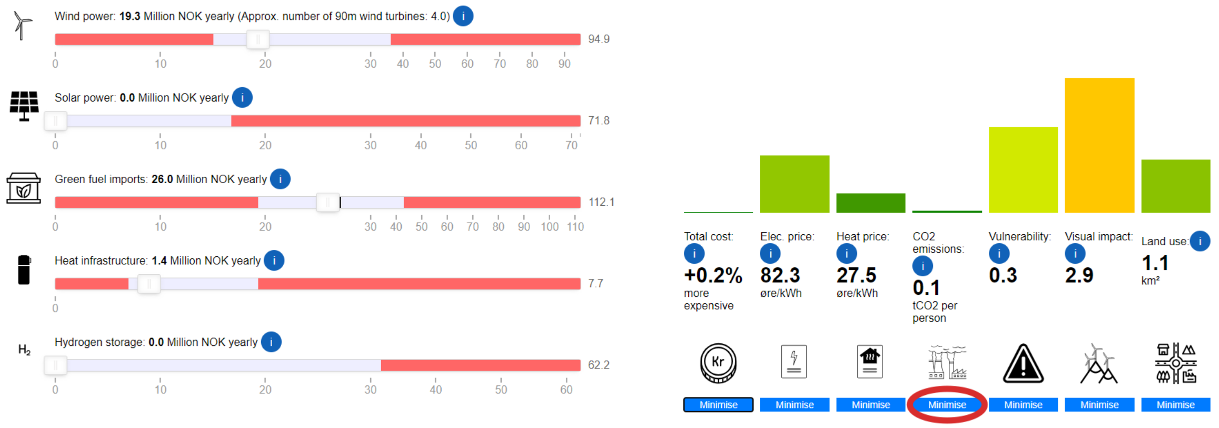

The resulting energy system does indeed lead to reduced CO_2 emissions, but the system is both expensive and has a high vulnerability.

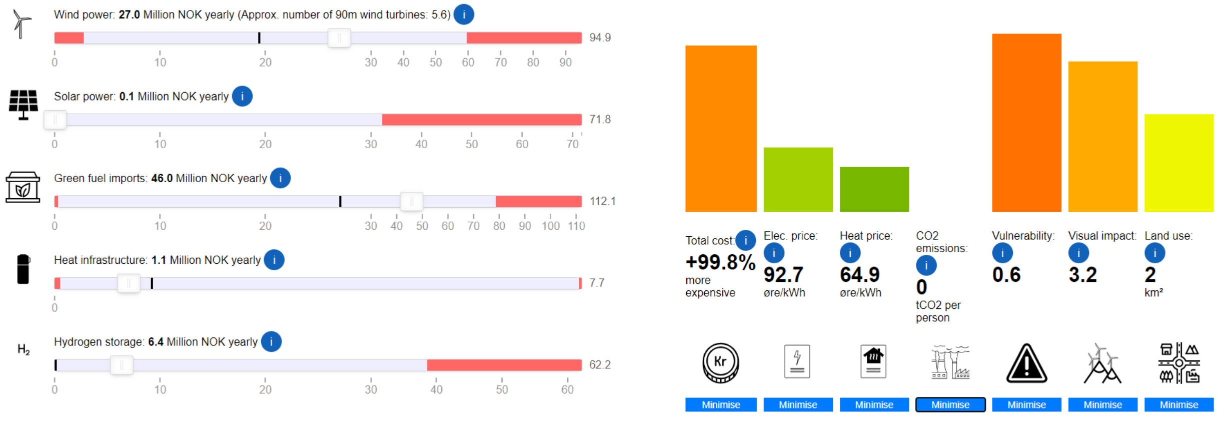

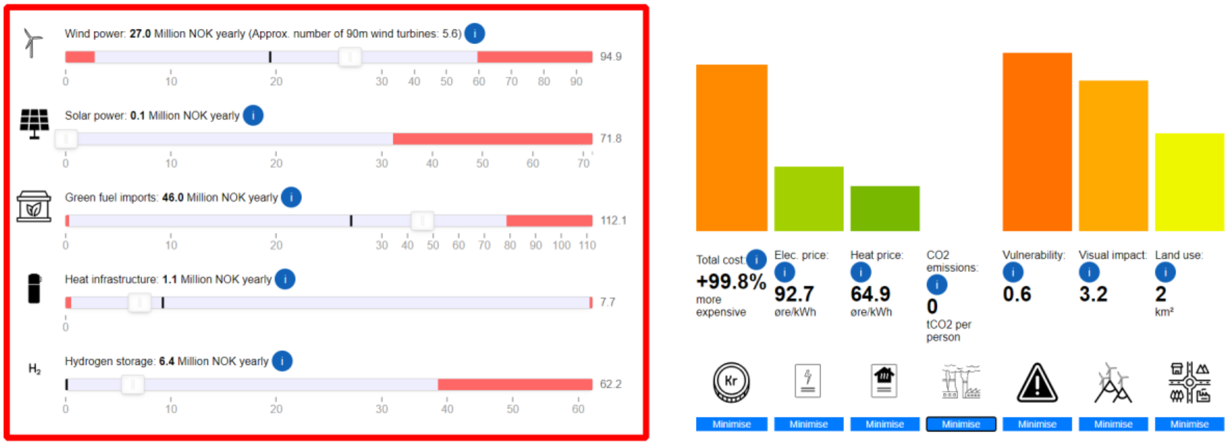

From this starting point, the participant continue to adjust the five sliders, to find a system that better balance their different priorities.

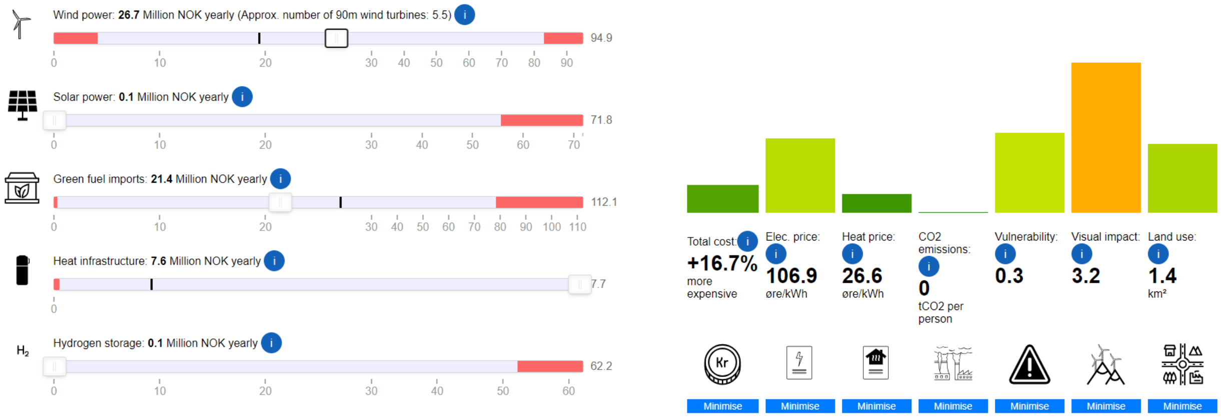

After some tweaking, the participant have found a system design with low CO_2 emissions, an acceptable level of vulnerability and 16.7% higher total system cost than the least-cost solution. The participant may then feel happy with their identified system and decide to finish there.

References

1. Allen, P., and Chatterton, T. (2013). Carbon reduction scenarios for 2050: An explorative analysis of public preferences. Energy Policy *63*, 796–808. doi: `10.1016/j.enpol.2013.08.079`.
2. Demski, C., Spence, A., and Pidgeon, N. (2017). Effects of exemplar scenarios on public preferences for energy futures using the my2050 scenario-building tool. Nature Energy *2*, 1–7. doi: `10.1038/nenergy.2017.27`.
3. Pidgeon, N., Demski, C., Butler, C., Parkhill, K., and Spence, A. (2014). Creating a national citizen engagement process for energy policy. Proceedings of the National Academy of Sciences *111*, 13606–13613. doi: `10.1073/pnas.1317512111`.
4. Xexakis, G., and Trutnevyte, E. (2019). Are interactive web-tools for environmental scenario visualization worth the effort? An experimental study on the Swiss electricity supply scenarios 2035. Environmental Modelling & Software *119*, 124–134. doi: `10.1016/j.envsoft.2019.05.014`.
5. Xexakis, G., Hansmann, R., Volken, S.P., and Trutnevyte, E. (2020). Models on the wrong track: Model-based electricity supply scenarios in Switzerland are not aligned with the perspectives of energy experts and the public. Renewable and Sustainable Energy Reviews *134*, 110297. doi: `10.1016/j.rser.2020.110297`.

6. Volken, S.P., Xexakis, G., and Trutnevyte, E. (2018). Perspectives of Informed Citizen Panel on Low-Carbon Electricity Portfolios in Switzerland and Longer-Term Evaluation of Informational Materials. Environmental Science & Technology *52*, 11478–11489. doi: 10.1021/acs.est.8b01265.

7. Holzer, S., Dubois, A., Cousse, J., Xexakis, G., and Trutnevyte, E. (2023). Swiss electricity supply scenarios: Perspectives from the young generation. Energy and Climate Change *4*, 100109. doi: 10.1016/j.egycc.2023.100109.

8. Dubois, A., Holzer, S., Xexakis, G., Cousse, J., and Trutnevyte, E. (2019). Informed Citizen Panels on the Swiss Electricity Mix 2035: Longer-Term Evolution of Citizen Preferences and Affect in Two Cities. Energies *12*, 4231. doi: 10.3390/en12224231.

9. Flacke, J., and De Boer, C. (2017). An Interactive Planning Support Tool for Addressing Social Acceptance of Renewable Energy Projects in The Netherlands. ISPRS International Journal of Geo-Information *6*, 313. doi: 10.3390/ijgi6100313.

10. Mayer, L.A.F., Bruine de Bruin, W., and Morgan, M.G. (2014). Informed Public Choices for Low-Carbon Electricity Portfolios Using a Computer Decision Tool. Environmental Science & Technology *48*, 3640–3648. doi: 10.1021/es403473x.

11. Department for Energy Security and Net Zero (2024). Carbon calculator. https://www.gov.uk/guidance/carbon-calculator. .

12. Berntsen, P.B., and Trutnevyte, E. (2017). Ensuring diversity of national energy scenarios: Bottom-up energy system model with Modeling to Generate Alternatives. Energy *126*, 886–898. doi: 10.1016/j.energy.2017.03.043.

13. Neumann, F., and Brown, T. (2021). The near-optimal feasible space of a renewable power system model. Electric Power Systems Research *190*, 106690. doi: 10.1016/j.epsr.2020.106690.
